# Trends in Recurring and Chronic Food Insecurity Among US Families With Older Adults

**DOI:** 10.1001/jamahealthforum.2023.5463

**Published:** 2024-03-01

**Authors:** Cindy W. Leung, Noura E. Insolera, Julia A. Wolfson

**Affiliations:** 1 Department of Nutrition, Harvard T.H. Chan School of Public Health, Boston, Massachusetts; 2 Institute for Social Research, University of Michigan, Ann Arbor; 3 Department of International Health, Johns Hopkins Bloomberg School of Public Health, Baltimore, Maryland; 4 Department of Health Policy and Management, Johns Hopkins Bloomberg School of Public Health, Baltimore, Maryland

## Abstract

**Importance:**

Food insecurity is a critical social determinant of health for older adults. Understanding national food insecurity trends among families with older adults has important policy implications.

**Objective:**

To compare food insecurity trends among US families with an older adult from 1999 to 2003 and 2015 to 2019 and further stratify the analysis by race and ethnicity, socioeconomic status markers, and enrollment in the federal Supplemental Nutrition Assistance Program (SNAP).

**Design, Setting, and Participants:**

In this cohort study using biennial data from the nationally representative Panel Study of Income Dynamics, balanced panels of families with at least 1 older adult (≥60 years) who participated from 1999 to 2003 (n = 1311) and 2015 to 2019 (n = 2268) were created. Analysis was completed in 2023.

**Main outcome:**

Food insecurity was assessed using the US Household Food Security Survey Module. Within each 5-year period, we defined recurring food insecurity as 2 or more episodes of food insecurity and chronic food insecurity as 3 episodes of food insecurity.

**Results:**

Overall, food insecurity among US families with older adults increased from 12.5% in 1999 to 2003 to 23.1% in 2015 to 2019. Rates of recurring food insecurity more than doubled (5.6% to 12.6%), whereas rates of chronic food insecurity more than tripled (2.0% to 6.3%). Across both time periods, higher rates of food insecurity persisted among Black and Hispanic families, with lower socioeconomic status, and participating in SNAP.

**Conclusions and Relevance:**

These results highlight how rates of recurring and chronic food insecurity among families with older adults rose substantially over the past 20 years. Monitoring national trends in food insecurity among older adults has direct programmatic and policy implications.

## Key Points

**Question:**

How have food insecurity trends changed for older adults in the US?

**Findings:**

This cohort study found that food insecurity among US families with older adults increased substantially over 2 decades—from 1 in 8 families (12.5%) in 1999 to 2003 to 1 in 4 families (23.1%) in 2015 to 2019. Rates of recurring food insecurity more than doubled (5.6% to 12.6%), whereas rates of chronic food insecurity more than tripled (2.0% to 6.3%).

**Meaning:**

Food insecurity adversely affects virtually every health domain for older adults and it is critical to coordinate clinical and policy approaches to reduce food insecurity and its subsequent health risks to promote healthy aging.

## Teaser

This cohort study compares food insecurity trends among US families with older adults from 1999-2003 and 2015-2019 stratified by race and ethnicity, socioeconomic status markers, and enrollment in the federal Supplemental Nutrition Assistance Program.

## Introduction

Food insecurity is a critical social determinant of health for older adults.^1,2^ In 2021, food insecurity affected 5.5 million older adults (ages 60 years and older) in the US; by 2050, that estimate is projected to grow to more than 7 million older adults.^2^ Food insecurity is a dynamic process that ebbs and flows with macroeconomic and microeconomic factors, major life events, and health conditions. Understanding national food insecurity trends is important for informing nutrition, antihunger, and other social policies.

We compared food insecurity trends among US families with an older adult in recent years (2015-2019) with those from 20 years ago (1999-2003). We then stratified the analyses by race and ethnicity, socioeconomic status, and enrollment in the federal Supplemental Nutrition Assistance Program (SNAP).

## Methods

Data were obtained from the Panel Study of Income Dynamics (PSID), the longest running nationally representative household panel survey. Data collection protocols for the PSID are approved by the University of Michigan. Further institutional review board review was not needed as the present study used existing, publicly available data. Participants provided written informed consent. Data collection began in 1968 and has followed the original sample and their families annually until 1997, and biennially since 1997. PSID has collected data on food insecurity in 6 waves: 1999, 2001, 2003, 2015, 2017, and 2019. Balanced panels of families with at least 1 householder aged 60 years or older who participated in all 3 waves of PSID from 1999 to 2003 (n = 1311) and 2015 to (n = 2268) were compared. The study followed the Strengthening the Reporting of Observational Studies in Epidemiology (STROBE) reporting guidelines.

Food insecurity was assessed using the USDA Household Food Security Survey Module (HFSSM).^3^ Similar to prior studies, we defined food insecurity as 1 or more affirmative response to any of the questions.^4,5^ We then tabulated the number of waves, from 0 to 3, in which a family experienced food insecurity between 1999 and 2003 and 2015 and 2019. Within each time period, we defined *any food insecurity* as food insecurity in at least 1 biennial survey wave; *recurring food insecurity* as food insecurity in at least 2 biennial survey waves; and *chronic food insecurity* as food insecurity in all 3 biennial survey waves. These categories are not mutually exclusive; for example, a family with chronic food insecurity would also be categorized as having recurring food insecurity and any food insecurity. This approach to categorizing the number of survey waves with food insecurity aligns with a prior study^6^ of US families with children.

We further evaluated differences in food insecurity trends by race and ethnicity and educational attainment of the PSID reference person, and by total income and SNAP enrollment of the PSID family. Race and ethnicity were self-reported by the PSID reference person using categories based on the US Office of Management and Budget Standards. For the present analysis, we focused stratified analyses on Hispanic, non-Hispanic Black (hereafter Black), and non-Hispanic White (hereafter White) families because fewer than 2% of the PSID sample identified as Asian, Native Hawaiian or Pacific Islander, or American Indian or Alaska Native. Longitudinal family-level survey weights, cluster, and strata accounted for the original sample design, attrition and death, and to make nationally representative estimates for families with at least 1 householder aged 60 years or older.

## Results

**Figure 1.**
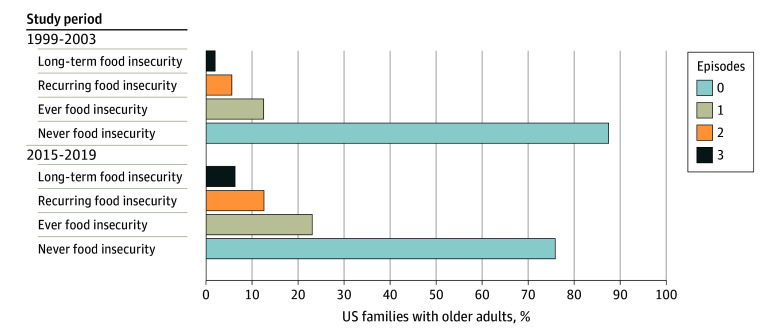
Trends in Food Insecurity Among US Families With Older Adults by Race and Ethnicity From 1999 to 2003 and 2015 to 2019 (Shown as Weighted Proportions)

Figure 1 shows overall trends in food insecurity among US families with older adults between 1999 and 2003 and 2015 and 2019. Between 1999 and 2003, 12.5% of families with an older adult had experienced any food insecurity. Between 2015 and 2019, 23.1% of families with an older adult had experienced any food insecurity—a 1.85-fold increase. Furthermore, the prevalence of recurring food insecurity (ie, food insecurity in ≥2 survey waves) more than doubled from 5.6% in 1999 to 2003 to 12.6% in 2015 to 2019, and the prevalence of chronic food insecurity (ie, food insecurity in all 3 survey waves) more than tripled from 2.0% in 1999 to 2003 to 6.3% in 2015 to 2019.

Increasing trends in all food insecurity categories were observed among Black, Hispanic. and White families with older adults over the 20-year period (Table). Among Black families, recurring food insecurity (ie, food insecurity in 2 or more survey waves) rose from 22.5% to 26.8%; chronic food insecurity (ie, food insecurity in all 3 survey waves) rose from 8.9% to 12.8%. Among Hispanic families, recurring food insecurity nearly doubled from 17.2% to 34.3%; chronic food insecurity more than doubled from 9.0% to 18.7%. At both time points, food insecurity levels were substantially higher among Black and Hispanic families than White families. However, among White families, chronic food insecurity rose nearly 6-fold, from 0.8% between 1999 and 2003, to 4.5% between 2015 and 2019.

**Table.** Trends in Food Insecurity Among US Families With Older Adults by Race and Ethnicity and Markers of Socioeconomic Status From 1999 to 2003 and 2015 to 2019 (Shown as Weighted Proportions)

Variable	1999-2003	2015-2019	Ratio
**Non-Hispanic Black families with an older adult**			
Any food insecurity (≥1 episode)	36.8	41.3	1.12
Recurring food insecurity (≥2 episodes)	22.5	26.8	1.19
Chronic food insecurity (3 episodes)	8.9	12.8	1.44
**Hispanic families with an older adult**			
Any food insecurity (≥1 episode)	40.0	49.7	1.24
Recurring food insecurity (≥2 episodes)	17.2	34.3	1.99
Long term food insecurity (3 episodes)	9.0	18.7	2.08
**Non-Hispanic White families with an older adult**			
Any food insecurity (≥1 episode)	8.6	18.4	2.14
Recurring food insecurity (≥2 episodes)	3.3	8.8	2.67
Chronic food insecurity (3 episodes)	0.8	4.5	5.63
**Families whose respondent did not attend college**			
Any food insecurity (≥1 episode)	16.5	31.1	1.88
Recurring food insecurity (≥2 episodes)	7.9	18.3	2.32
Chronic food insecurity (3 episodes)	2.7	8.8	3.26
**Families with incomes ≤130% of the federal poverty level**			
Any food insecurity (≥1 episode)	29.5	52.7	1.79
Recurring food insecurity (≥2 episodes)	17.4	34.5	1.98
Chronic food insecurity (3 episodes)	7.7	18.8	2.44

**Figure 2.**
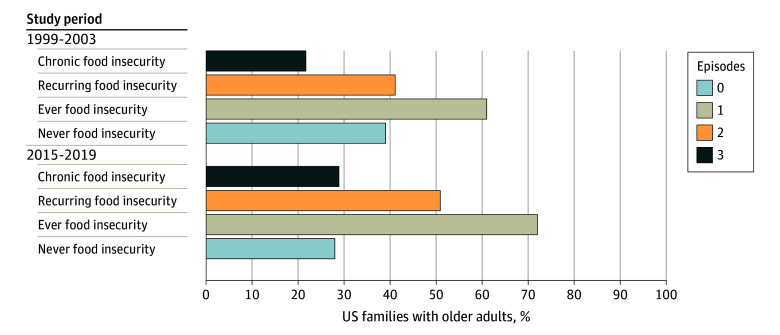
Trends in Food Insecurity Among US Families With Older Adults Enrolled in the Supplemental Nutrition Assistance Program From 1999 to 2003 and 2015 to 2019 (Shown as Weighted Proportions)

At both time points, food insecurity disproportionately affected families with older adults with low educational attainment (ie, no college attendance) and low incomes (ie, ≤130% of the federal poverty level) (Table). Among families with low education, recurring food insecurity (ie, food insecurity in ≥2 survey waves) increased from 7.9% to 18.3%, and chronic food insecurity (ie, food insecurity in all 3 survey waves) increased from 2.7% to 8.8%. Among families with low incomes, recurring food insecurity increased from 17.4% to 34.5%, and chronic food insecurity increased from 7.7% to 18.8%. Among families participating in SNAP, 61% had any experience of food insecurity between 1999 and 2003, which rose to 72% between 2015 and 2019. Similarly, recurring food insecurity increased from 41.1% to 50.9% and chronic food insecurity increased from 21.7% to 28.9% (Figure 2).

## Discussion

To our knowledge, this is the first longitudinal study to compare national trends in food insecurity among US families with older adults over 2 decades. These results highlight 4 main points. First, overall food insecurity among families with older adults increased substantially during the 2 decades—from 12.5% in 1999 to 2003 to 23.1% in 2015 to 2019. Second, all categories of food insecurity increased between the 2 time periods across all racial and ethnic and socioeconomic subgroups. Third, the overall rate of chronic food insecurity among families with older adults more than tripled over the 20-year period, indicating that more families with older adults were becoming food insecure, and families with existing food insecurity were remaining food insecure. Finally, chronic food insecurity was disproportionately higher among families of color, with low incomes, and those participating in SNAP. However, White families and families with low educational attainment experienced the sharpest increases in chronic food insecurity over the 2 decades. These rising trends are concerning given that food insecurity is negatively associated with virtually every health domain among older adults.^4,7,8,9,10^ It is critical to coordinate clinical and policy approaches to reduce food insecurity and its subsequent health risks.

There have been increasing efforts to screen and address food insecurity in clinical settings.^1^ Although several interventions have targeted pediatric populations, these programs (eg, food referrals, produce prescriptions) should be expanded to geriatric medicine. Simultaneously, clinicians should receive training to hold sensitive discussions on food insecurity and other barriers to nutritious food with their aging patients.^11^ Furthermore, partnerships between health care systems and community organizations to provide medically tailored meals can serve as a promising model to integrate nutrition assistance, social support, and health care services for home-bound older adults.^12^

In the policy arena, SNAP is the largest program to address food insecurity, which reaches 5.3 million households with older adults.^13^ Results from the present study demonstrate that the proportions of households receiving SNAP who experienced ever, recurring, or chronic food insecurity were substantially greater than the overall population, and at levels that widened across the 2 decades. Although this indicates that SNAP is effective at targeting those households most vulnerable to food insecurity, it also suggests that SNAP could be strengthened to better alleviate food insecurity.^14^ The recent changes to SNAP during the COVID-19 pandemic provide a natural experiment to examine which policy changes were most effective at reducing food insecurity among older adults.

### Limitations

This study has limitations. PSID did not assess food insecurity between 2005 and 2013, so the extent to which families transitioned in and out of food insecurity during that period is unknown. PSID surveys are administered biennially and we cannot determine the state of food insecurity during the in-between years. Food insecurity indicators were assessed over the previous 12 months, which may lead to underreporting of food insecurity due to recall bias or social desirability bias.^15^ Lastly, we were unable to compare food insecurity trends in families identifying as Asian, Native Hawaiian or Pacific Islander, American Indian or Alaska Native, and other minoritized racial groups due to very small sample sizes.

## Conclusions

The results of this cohort study of US families with older adults highlight how trends in all food insecurity categories rose substantially over the past 20 years, and particularly among Black and Hispanic families and families with low socioeconomic status. Future research should focus on the effect of the COVID-19 pandemic on food insecurity trends and identifying policy and programmatic strategies to reduce food insecurity among families with older adults.

## Notes

Supplement. Data Sharing Statement
