# Supplementary material for: Trends in Recurring and Chronic Food Insecurity Among US Families With Older Adults
Source: JAMA Health Forum. 2024 Mar 1;5(3):e235463. doi: 10.1001/jamahealthforum.2023.5463 (PMC10907918; doi:10.1001/jamahealthforum.2023.5463)
Supplement: Supplement. — Data Sharing Statement [file jamahealthforum-e235463-s001.pdf]

Data Sharing Statement

Leung. Trends in Recurring and Chronic Food Insecurity Among US Families With Older Adults. *JAMA Health Forum*. Published March 01, 2024.
doi:10.1001/jamahealthforum.2023.5463

Data
Data available: No

Additional Information
Explanation for why data not available: Data for the Panel Study of Income Dynamics is publicly available at psidonline.isr.umich.edu.
